# Carbohydrate Derived Organogelators and the Corresponding Functional Gels Developed in Recent Time

**DOI:** 10.3390/gels4020052

**Published:** 2018-05-30

**Authors:** Nabamita Basu, Arijit Chakraborty, Rina Ghosh

**Affiliations:** 1 Department of Chemistry, Nabagram Hiralal Paul College, Konnagar, West Bengal 712246, India; basu0nabomita@gmail.com; 2 Department of Chemistry, Acharya B. N. Seal College, Cooch Behar, West Bengal 736101, India; arijit_chak2002@yahoo.com; 3 Department of Chemistry, Jadavpur University, Kolkata 700032, India

**Keywords:** carbohydrates, organogelators, gels, sugars

## Abstract

Owing to their multifarious applicability, studies of molecular and supramolecular gelators and their corresponding gels have gained momentum, particularly in the last two decades. Hydrophobic–hydrophilic balance, different solvent parameters, gelator–gelator and gelator–solvent interactions, including different noncovalent intermolecular interactive forces like H-bonding, ionic interactions, π–π interactions, van der Waals interactions, etc., cause the supramolecular gel assembly of micro and nano scales with different types of morphologies, depending on the gelator, solvent, and condition of gelation. These gel structures can be utilized for making template inorganic superstructures for potential application in separation, generation of nanocomposite materials, and other applications like self-healing, controlled drug encapsulation, release and delivery, as structuring agents, oil-spill recovery, for preparation of semi-conducting fabrics, and in many other fields. Sugars, being easily available, inexpensive, and nontoxic natural resources with multi functionality and well-defined chirality are attractive starting materials for the preparation of sugar-based gelators. This review will focus on compilation of sugar derived organogelators and the corresponding gels, along with the potential applications that have been developed and published recently between January 2015 and March 2018.

## 1. Introduction

A wide variety of self-organized superstructures exist in almost every life form on earth. The spontaneous self-aggregation process plays a key role in the living organisms, and facilitates processes such as three-dimensional protein folding, DNA transcription and hybridization, cell membrane formation, and many more. Hence, the study of superstructure formation processes and their successful mimicking has drawn the attention of the scientific community for quite some time. Self-organization and supramolecular chemistry have also found an ever-growing significance in chemistry and physics, apart from the noticeable choice of biological sciences [1]. Supramolecular chemistry has been described by one of its leading exponents, Jean-Marie Lehn, as “the chemistry of molecular assemblies and of the intermolecular bond” and more informally expressed as “chemistry beyond the molecule” [2].

Gels are a wide variety of self-organized structures that have extended their use in human life in day-to-day usable products such as toothpaste, shampoo, soap, hair gel, shaving gel, honey, syrup, pudding, contact lenses, gel pens, and life-saving drug delivery systems. To put it simply, a ‘gel’ can be described as a self-organization of molecules in a 3D-network with the ability to trap the liquid phase with rheological parameters G’’/G’ having a value ≤0.1. Gels are the coexistence of gelators (solid phase) and a liquid solvent phase, the gelator part being responsible for forming the 3-D network [3], in which the solvent molecules are trapped and get immobilized. Gels can be classified into two categories, (i) chemical and (ii) physical gels depending upon the cross-linking among the molecules [4,5,6,7,8]. In chemical gels, the aggregation is governed by the formation of covalent cross-linkages of the compounds and leads to the formation of a thermally irreversible network [7]. Examples of these systems include cross-linked polymeric systems (e.g., polyamide, polyester, polyethylene, and poly vinyl alcohol) and may be natural or synthetic. Reports on polymeric thermoreversible gels were summarized early in 1992 in a book by Guenet [9]. Reversible physical polymeric gels are also known, when gels are obtained by the noncovalent interaction of polymer molecules. Polymer gels have been well-known for centuries for their diversified application in food, cosmetics, medicines, and other industrial and pharmacological products. On the other hand, if the three dimensional network of low molecular weight-derived compound is directed by a self-assembly process (by noncovalent interactions such as hydrogen bonding, π–π stacking, van der Waals interactions, electrostatic interactions, etc.), the resulting gel is termed a supramolecular or physical gel [4,5,6,7,8] and are usually thermally reversible.

Physical gel-forming small organic compounds are named as low molecular weight gelators or LMWGs [10]. LMWGs are a category of organic compounds with molecular mass less than 3000, which can be divided mainly into two classes depending on the polarity of the trapped solvent inside the 3D framework. These classes are (i) low molecular-weight organogelators or LMOGs (trapping organic solvents); and (ii) low molecular-weight hydrogels LMWHs (trapping water) [11,12]. Ambidextrous LMWGs are also well-known, and have the capability to bind both organic and aqueous solvents [13]. During the last two to three decades, studies on low molecular weight gelators and their corresponding gels have gained much interest, and several reviews have been published including various aspects of such gels [4,5,6,14,15,16,17,18,19,20,21,22,23,24,25,26,27], along with a recently published book by Guenet on organogels [28]. A few hydrogelators with the ability to gelate water in the presence of 1–10% dimethyl sulfoxide or methanol have already been reported [29]. But, there are only a few low molecular weight hydrogelators (LMWHs) that form gels in water or aqueous buffers without any additional organic solvents [30,31]. Low molecular-mass organogelators or LMOGs form in only specific organic media. The 3D superstructures are maintained by a subtle balance between the gelator–gelator interaction and gelator–solvent interaction [4]. In case of LMOGs, the gelation process is thermally reversible across the gel transition temperature, Tgel (the sol-gel transition temperature) [4]. Intermolecular interactions like π–π stacking, H-bonding, dipolar interaction, and London dispersion forces [32] are responsible for the stabilization of LMOG assemblies.

Apart from the mentioned interactive forces, hydrophobic–hydrophilic balance, different solvent parameters, viz. partition coefficient (logP), Henry’s law constant (HLC), Kamlet–Taft parameters (β, α, and π), solvatochromic parameters [ET(30)], and Hansen solubility parameters (δp, δd, and δh) greatly correlate with the gelation ability with several types of gelators and also may help to find a priori to congeal a particular solvent [33].

## 2. Carbohydrate Derived Low Molecular Weight Gelators

### 2.1. Importance of Carbohydrates as Renewable Resources in This Field

**Figure 1:**
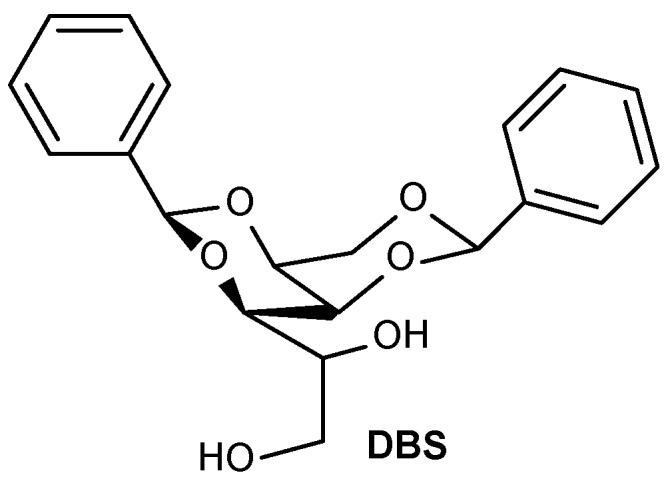
1,3:2,4-Dibenzylidene-*d*-sorbitol (DBS), **1**.

In nature, carbohydrates are the main sources of energy for living organisms, and glucose is the main source of energy for the metabolism of various life forms. This is also a product of photosynthesis, a process which plants and algae use for the conversion of sunlight into chemical energy for their survival. Among other important abundant sugars these may be made from polymers of glucose, e.g., glycogen in animals and starch in the plant kingdom, and can be used as an energy source, structuring polymer e.g., cellulose of plants and algae, DNA as genetic material, and RNA. Carbohydrates are readily available naturally abundant renewable resources containing different functional groups, some of which are having well defined chiral centers [34]. Because sugars contain multiple chiral centers, they are ideal for synthesizing compounds that are able to self-assemble into gel-like structures having chiral sense. Thus, the design and development of supramolecular soft materials using functional small molecules like carbohydrates is a rapidly growing research arena. For many years, unprotected sorbitol, a reduced form of glucose, was well-known to act as a thickener (albeit not formally a gelator) in water, and as a consequence of its rheological properties and sweetness, is exploited extensively in the food industry as well as being used for the formulation of cosmetic and other consumer products. The first report of carbohydrate based organogelators dates was back to 1891 when Meunier reported the gelation phenomenon of two benzaldehyde-*d*-sorbitols, produced when a mixture of *d*-sorbitol and benzaldehyde was condensed with strong mineral acids [35]. About fifty-three years later, the chemical structure was proved by another group to be 1,3:2,4-dibenzylidene-*d*-sorbitol (DBS, **1**, Figure 1) [36].

Carbohydrate-based gelation gradually became a topic of interest with phenomenal research from Yamasaki [37], Hasoda [38], Watanabe [39], Shimizu [40,41], Nolte [42], and most importantly, with the pioneering work of Shinkai [43,44,45,46,47,48]. For the last two decades gelators based on carbohydrate-derived low molecular-weight compounds have gained the interest of several research groups. The formation of low molecular-weight gelators from sugar derivatives has potential impacts in advanced materials and supramolecular chemistry. The intrinsic chirality and biocompatibility of sugar-derived self-assembling systems have many special applications, such as separating biomolecules, forming liquid crystals, use in optical devices and controlled drug delivery systems [49,50], and many others. Due to the presence of a large number of hydroxyl groups, carbohydrates have been proven to be the obvious choice for organogelators with gelation properties manifested via H-bonding. Recently, Bhattacharya and coworkers compiled the carbohydrate derived gelators and their corresponding gels in a review, published in 2015 [27], where they elaborately discussed the multifarious facets of sugar-gels, including their gelation mechanisms and different applications. In the present review we will focus only on the assemblage of the carbohydrate-based organogels and their applications, published in the period between January 2015 and March 2018.

### 2.2. Gelators Utilizing Renewable Sugar Resources

#### 2.2.1. Gelators Based on Alditols

Since the first report by Meunier [35] on a DBS gel a lot of reports based on this renewable resource have been made, including a recent review in 2015 [51]. Recently, turbid gels in ionic liquids and transparent gels of acetophenone have been reported based on the DBS gelator. Gels were analyzed by Synchrotron small-angle X-ray scattering (SAXS) and polarized optical microscopy [52]. Recently, Lai et al. [53] observed that DBS molecules in a solution of low molecular weight polyethylene glycol (PEG), self-assemble, finally congealing PEG. This group has studied the effect of inorganic silica on such hybrid organogel. Silica affects the intermolecular H-bonding between DBS and PEG, probably due to its increasing interaction with PEG, which, in effect, increases the self-assembly of DBS molecules; T_gel_ of the silica modified hybrid organogel increases.

Lai et al. have also used hydrophobic poly(vinylidene)fluoride [PVDF] [54] as a polymer matrix for studying the effect on morphology formation of self-assembled nanofibrils based on another sorbitol derivative, 1,3:2,4-di(3,4-dimethylbenzylidene)sorbitol [DMDBS] upon heat treatment; this exhibited different behavior compared to that based on the DBS/Poly(*l*-lactic acid) composite system reported previously [55]. Recently, in order to investigate the role of π–π stacking and H-bonding during the gel assembly of DBS, Rogers and coworkers synthesized analogues of DBS, viz. 1,3:2,4-dicyclohexylidene- and 1,3:2,4-diethylidene-*d*-sorbitols, which are devoid of an aromatic moiety, and also acetylated DBS, that is devoid of free OH [56]. They have established that the π–π stacking takes part in the formation of self-assembled fibrillar networks (SAFiNs) during gel assembly.

Other derivatives of sorbitol have also been synthesized toward exploration of gelation. Pal and coworkers [57], have utilized sorbitan monostearate (SMS) for the formation of a biocompatible organogel of sesame oil. The organogel was characterized by phase contrast microscopy, IR, DLS experiments, and also by rheological experiments. Later, Pal et al. also studied the effect of the concentration of this sorbitan monostearate (Span 60) on the mechanical and thermal properties [58].

**Figure 2:**
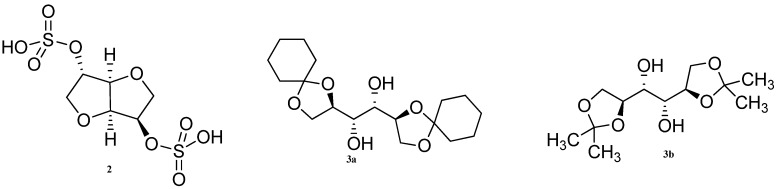
Isosorbide based gelator **2** and mannitol derived gelators **3a** and **3b**.

Three new gelators (**2**, **3a**,**b**, Figure 2) were prepared from isosorbide and mannitol derivatives, which formed gels with 21 different ionic liquids [59]. They have also studied the effect of cations and anions in the ionic liquids. They have established that the gelation ability of the ionic liquids can be correlated with Kamlet–Taft parameter of the ionic liquids, and H-bonding ability of the ionic liquids influence greatly in gel formation.

**Figure 3:**
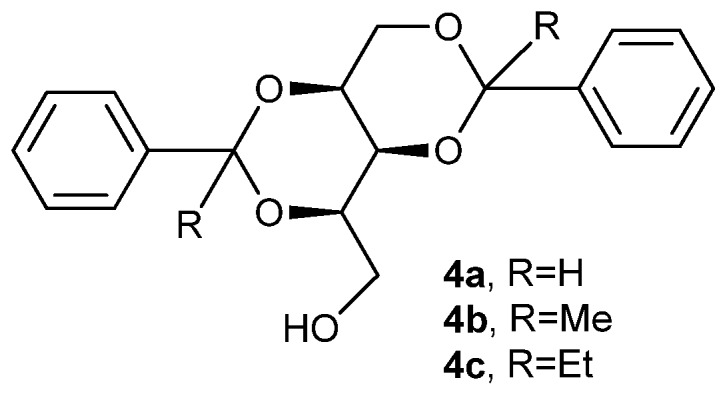
Xylitol based di-*O*-benzylidene acetal, **4a** and ketals, **4b**,**c**.

Apart from sorbitol, mannitol and xylitol have also been exploited as naturally inexpensive resources. Raju et al. have prepared the xylitol-based benzylidene acetal **4a** and two ketals (**4b**,**c**, Figure 3) which congeal organic solvents including crude fuel oils and vegetable oils [60]. The gelation ability of the gels in different nonpolar solvents and oils, based on gelators **4a–c**, decreased with increasing hydrophobicity and steric bulk of the protecting groups, as evidenced from Table 1. IR studies and SEM images indicated H-bonding to be responsible for the fibrillar gel networks.

#### 2.2.2. Gelators Based on Monosaccharides and Disaccharides

Yadav and coworkers have recently shown that arabinose-based partially-protected esters were able to congeal aromatic solvents as well as petrol and diesel [61], and that H-bonds between the OH and ester C=O are primary interactions towards gelation, as established by IR and wide-angle X-ray scattering. In the 1,2-dibenzoylated arabinose system π-interactions play an important role, causing more regular arrangement and better mechanical properties compared to those of the corresponding acetyl derivatives.

Shinkai and coworkers have synthesized several 4,6-*O*-arylidene derivatives of D-Glc, D-Gal, and D-Man under mild condition in one-step, using aromatic aldehydes in the presence of triethylorthoformate and cat. pTSA [62]. Several of these compounds form organogels with a variety of solvents, and two of these derivatives form hydrogels. Of the gelators, methyl 3-*n*-butoxybenzylidene-α-*d*-glucopyranoside formed a clear organogel and opaque hydrogel, both of which are thixotropic. The concentration of CGC of this gelator in squalane was 0.02 wt % (one of the lowest values for an oil gelator). Kowalczuk has chosen a methyl 4,6-*O*-(*p*-nitrobenzylidene)-α-*d*-glucopyranoside-toluene gel for the determination of different structural parameters of the gel matrix [63].

**Figure 4:**
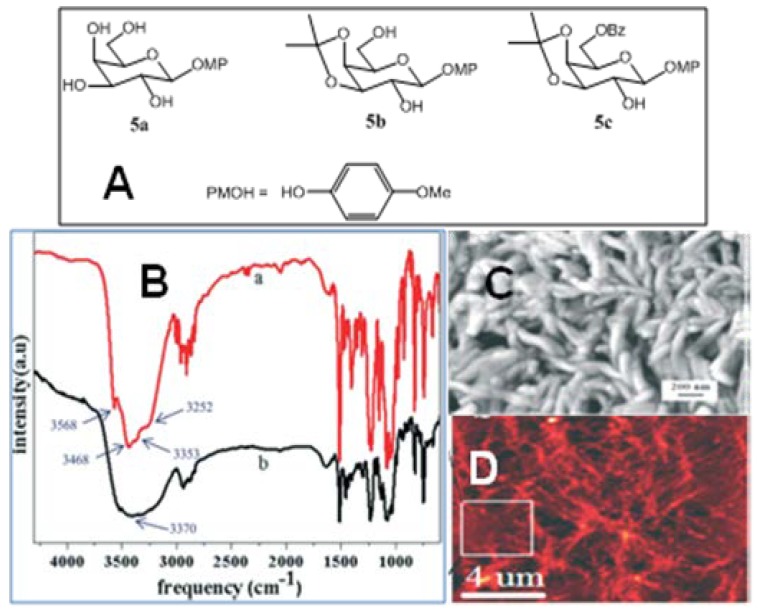
(**A**) Structures of the sugar compounds; (**B**) FT-IR spectra of compound **5a** in (a) crystalline state and (b) xerogel state (0.25% *w*/*v* in 1,2-dichlorobenzene); (**C**) Field emission scanning electron microscopy (FE-SEM) image of the gel of **5a** in 1,2-dichlorobenzene at their CGCs, the scale bar is 200 nm; (**D**) AFM images of the gels of **5a** in 1,2-dichlorobenzene. Reprinted with permission from [64]. Copyright 2015 Royal Society of Chemistry.

From the XRD study of the crystals, XPRD of the xerogels, and IR experiments, Mukhopadhyay and coworkers have made a correlation between gelling/nongelling behavior and the presence (or absence) of a one dimensional (1D) hydrogen-bonded network in single crystal structures of three galactose derivatives viz. *p*-methoxyphenyl β-*d*-galactopyranoside (**5a**), *p*-methoxyphenyl 3,4-*O*-isopropylidene-β-*d*-galactopyranoside (**5b**) and *p*-methoxyphenyl 6-*O*-benzoyl-3,4-*O*-isopropylidene-β-*d*-galactopyranoside (**5c**) (Figure 4) [64]. According to previous such correlation hypotheses [16,65], the presence of a 1D hydrogen-bonded network was suggested to promote efficient gelation in hydrogen bond-based gelators, and those with a 0D, 2D, or 3D crystal network are supposed to be poor gelators. Unexpectedly, saccharide **5a**, having a 2D hydrogen-bonded network (HBN), showed efficient gelation ability in various solvents, while saccharide **5c**, with a 1D HBN, was a nongelator. The authors believed that the failure of the latter could be due to the inappropriate surface compatibility of self-assembled fibrillar networks and the concerned solvent molecules. On the other hand, saccharide **5b**, with a 2D HBN, was found to be a nongelator, as expected. Importantly, easily prepared and eco-friendly LMOG saccharide **5a**, is an efficient gelator of 1,2-dichlorobenzene, with a critical gelation concentration of only 0.25% *w*/*v*. FT-IR spectroscopy showed the involvement of intermolecular hydrogen bonding in the gelation process (Figure 4B), and its true gel behavior was confirmed by rheological experimental results. SEM and AFM studies (Figure 4B,C) revealed the fibrous network of the molecules in the gel state.

**Figure 5:**
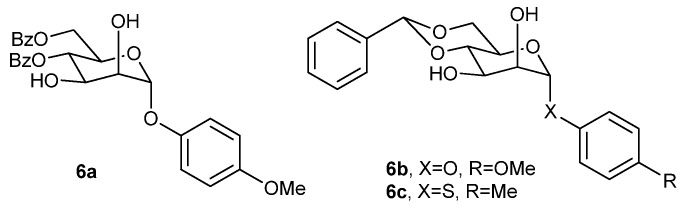
Mannose derived multifunctional low molecular-mass organic gelators (LMOGs).

Mukhopadhyay and coworkers also established that a mannose derived multifunctional LMOG (**6a**, Figure 5) can form an excellent gel in different organic solvents and oils at low concentration [66]. The gel based on **6a** exhibited recharge ability for up to four cycles of the burning-gelation process. Moreover, two other mannose-based gelators (**6b**,**c**, Figure 5), prepared by this group, were also capable of gelling several organic solvents and oils [67]. These gels show near-opacity in the UV-region, and thus may have future UV-screening application.

**Figure 6:**
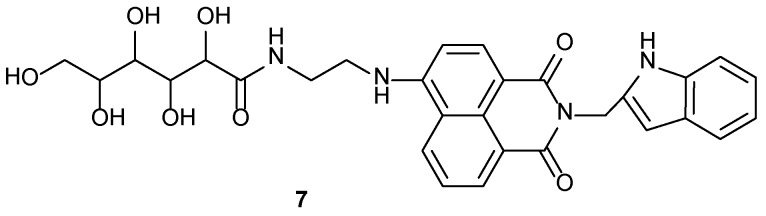
Sugar appended tryptamine derivative **7**.

Sugar appended tryptamine based gelator (**7**) (Figure 6) was designed and synthesized by Xu et al. [68]. Gelator **7** could gel various alcohols, accelerated by the application of heat and sonication. An interesting study on the morphology transformation of the MeOH-gel by application of heat or sonication indicated a vesicle to ribbon-like 3D gel network. The mechanism of this transformation was also established by IR-, VIS-, 1H NMR-, and fluorescence spectroscopies, and also by X-ray diffraction studies.

Methyl 3,4-di-*O*-benzyl-2-acetamido-2-deoxy-α-*d*-glucopyranoside was synthesized in 4 steps starting from *d*-GlcNAc by Narayana et al. [69]. This compound can gelate aromatic organic solvents and oils, with DMSO and EtOH forming the corresponding organogels, and hydrogels based on aqueous EtOH. The hydrogel obtained was used for the preparation of gold and silver nanoparticles, where sugars were caping agents of the nanoparticles. A toluene gel was used for the removal of a dye (Rhodamine B) from the aqueous solution.

**Figure 7:**
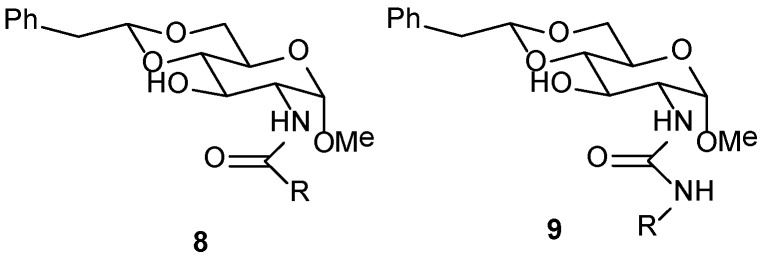
*d*-glucosamine derived amides **8** and ureas **9**.

*d*-glucosamine has also been used as a natural resource by Chen et al. [70] for preparation of several 4,6-*O*-benzylidene- and alkylidene-protected amides, and their corresponding urea analogs (**8** and **9**, Figure 7). These could gelate toluene, EtOH, iPrOH, ethylene glycol, and aqua-organic solvents. Of these, the amides are more effective gelators for pump oils and engine oil. Molecular assembly and the intermolecular forces were studied by ^1^H NMR spectroscopy. They also studied toluidine blue dye binding with one of the gelators appended with a naphthoyl group at the nitrogen.

**Figure 8:**
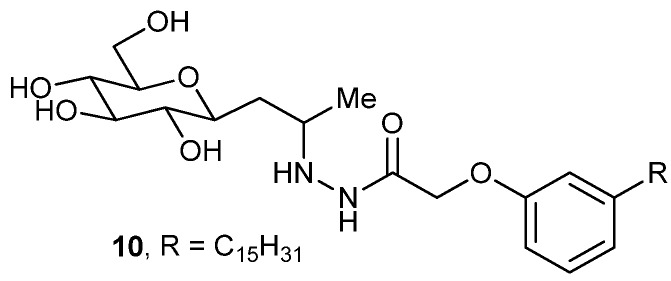
*d*-glucose based glycolipids.

Several glucose-based glycolipids have been synthesized, one compound (**10**, Figure 8) of this series forms a gel in aqueous–organic solvents like aqueous DMSO, aqueous DMF, aqueous EtOH, and aqueous MeOH (each in a 5:1 ratio) [71]. The gel morphologies using field emission scanning electron microscopy (FESEM) and field emission transmission electron microscopy (FETEM) techniques were based on the xerogel obtained from aqueous EtOH gel and showed twisted fibrous gel aggregates with lengths of 100–200 nm. The gel was further characterized by CD, FT-IR, and XRD analysis. The latter two revealed that H-bonding and π–π stacking are responsible for the gel assembly.

**Figure 9:**
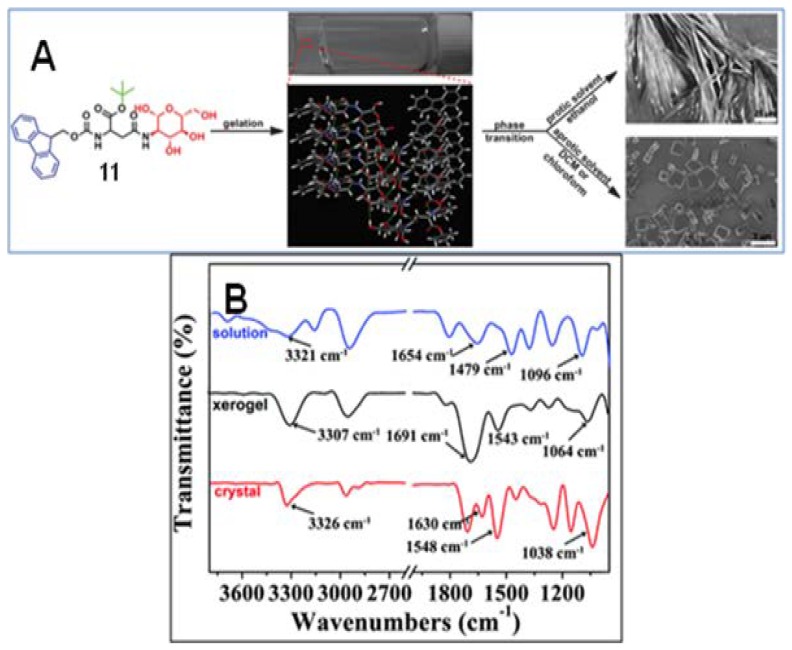
(**A**) Illustration of the self-assembly of Fmoc-Asp(Glc)-OtBu (**11**) in 1D microfibers and 3D microcubes in both protic and aprotic solvents; (**B**) FTIR analysis of the xerogel, microcrystals and solution in dichloromethane (DCM) (1.5 wt %). Reprinted with permission from [72]. Copyright 2016 Royal Society of Chemistry.

A new gelator (**11**, Figure 9A) was synthesized from *d*-GlcNH_2_ and FMocAsp with one of the carboxylic acid groups being a tert-butyl ester [72]. This molecule, having multifunctional groups, formed a supramolecular gel in protic and aprotic solvents. To understand the gelation process, gel characterization was done by rheological experiments, by FT-IR and XRD-analysis and also by molecular modeling studies. Comparison of the IR spectra (Figure 9B) of a solution of **11** in dichloromethane, of the xerogel, and of the microcrystals indicated that multiple H-bonding (evidenced by the shifting of the IR bands at 3328, 1657, and 1536 cm^−1^, corresponding to the non-H-bonded NH, amide I and amide II band, respectively, in dichloromethane, to other values compared to those of the xerogels and crystals), π–π stacking, and van der Waals interactions, were responsible for the gel- and also crystal-assembly. Interestingly, in CHCl_3_, EtOH, and MeOH the gel phase undergoes transformation into a crystal (Figure 9A), driven by unbalanced gelator–gelator and solvent–gelator interactions.

**Figure 10:**
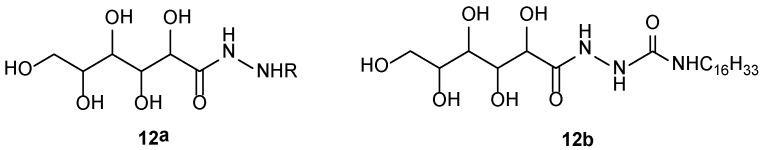
Gluconohydrazide **12a** and gluconosemicarbazide **12b**.

Gluconohydrazide (**12a**) and gluconosemicarbazide (**12b**, Figure 10) were synthesized by Himabindu et al. starting with a δ-gluconolactone [73]. Between these two, the semicarbazide was a more efficient gelator of DMSO-H_2_O (4:1, *v*/*v*), whether induced by heat or ultrasound. Interestingly, gels obtained by these two stimuli showed, to some extent, different morphologies as evidenced by electron microscopy studies. Comparison of their IR bands indicated a greater extent of H-bonding in the ultrasound-modulated gel. These two thermoelastic gels were stable in the presence of 0.05 M KCl, NaCl, CaCl_2_, and sodium dodecyl sulfate (SDS).

**Figure 11:**
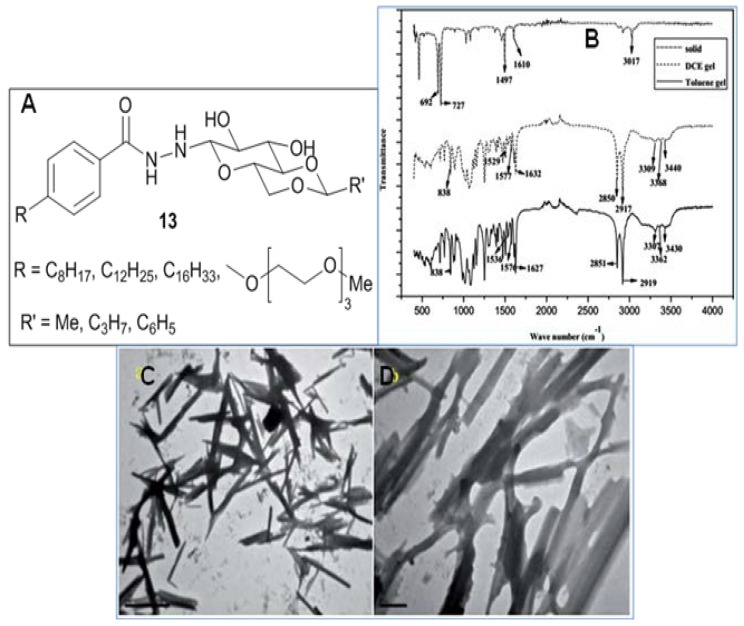
(**A**) Structures of gelators; (**B**) FTIR spectra of compound **13** (with R = OC_8_H_17_; R’ = CH_3_) solid, gel (dichloroethane), gel (toluene); high-resolution transmission electron microscopy (HR-TEM) images of this compound (dissolved in dichloroethane) under different magnifications: (**C**) 1 mm and (**D**) 0.5 mm. Reprinted with permission from [74]. Copyright 2016 Royal Society of Chemistry.

Twelve benzohydrazides (**13**, Figure 11A) based on 4,6-*O*-alkylidene/arylidene-*d*-Glcp were designed and synthesized by Mohan Das and coworkers [74]. Gelation of polar (1,2-dichlorobenzene and CHCl_3_) and nonpolar (1,2-dichloroethane, toluene and benzene) solvents has been described. Spherical and rod structures were evidenced by electron microscopic techniques (FE-SEM); powder XRD, and IR experiments (Figure 11B) suggested that H-bonding and van der Waals interactions were responsible for the gel assembly. TEM images showed thin and lengthy rods (Figure 11C,D) of **13** dissolved in dichloromethane.

#### 2.2.3. Gelators Incorporating the Triazole Moiety to Sugar by Click Chemistry

**Figure 12:**
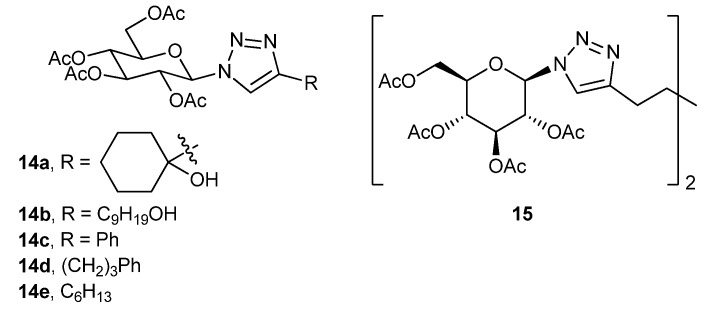
Glucosylated triazole derivatives.

A copper-catalyzed click reaction has been utilized for synthesis of a series of glucosyl triazole-derivatives (**14a–e**, **15**, Figure 12), and their supramolecular gel-assembly has been studied by electron microscopy, as well as by gelation in different solvents and rheological experiments by Wang’s research group [75]. Compounds containing hydroxyalkyl and aryl substituents on the triazole moiety are better gelators.

**Figure 13:**
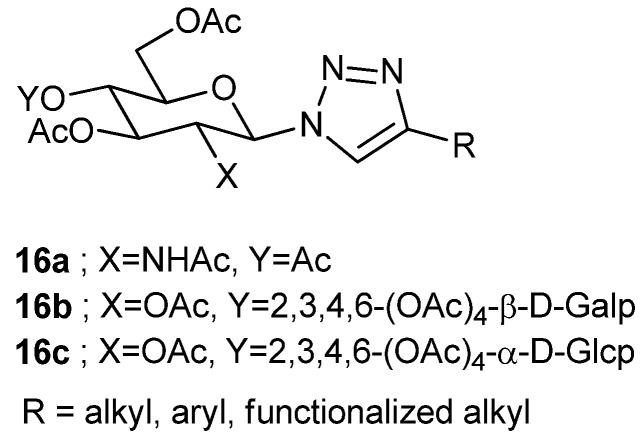
Mono- and di-saccharide derived glycosyl triazoles.

In a similar way, Wang et al. prepared several triazole per-*O*-acetyl-2-acetamido-2-deoxy-β-*d*-glucopyranoside derivatives (**16a**, Figure 13) with different alkyl, aryl/*N*- or, *O*-functionalized alkyl substituents on the triazole ring, some of which were organogelators, some were hydrogelators; many of these can congeal aqueous–organic solvents [76].

Wang’s group has also synthesized disaccharide derived amphiphiles [77]. Nine per-*O*-acetyl lactosyl- (**16b**, Figure 13) and thirteen per-*O*-acetyl maltosyl triazole-derivatives (**16c**, Figure 13) were prepared utilizing click chemistry. One of the lactosyl, and most of the maltosyl triazole compounds, were organogelators of aqueous DMSO (1:1) and aqueous EtOH (1:1). ^1^H-NMR of a model gel indicated that triazole ring and H-bonding were responsible for gel assembly. Morphology of the gel, rheology, and effect of alkyl and aryl substituents on the triazole moiety were also studied. Alkyl and aryl groups on the triazol part exhibited enhanced gelation.

**Figure 14:**
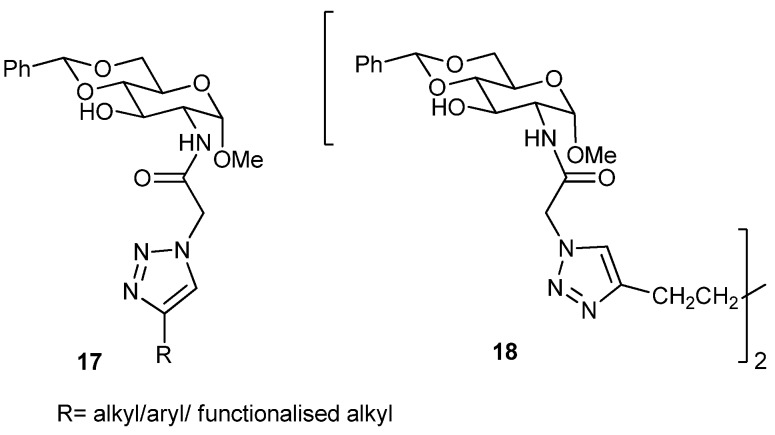
Triazolyl anchored *d*-glucosamide based glycolipids.

**Figure 15:**
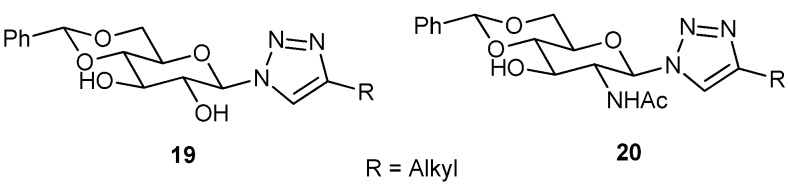
*d*-Glucose and *N*-acetylglucosamine derived triazoles.

Eighteen other novel organogelators (**17**, **18**, Figure 14), containing 4,6-*O*-benzylidenated *d*-glucosamide anchored with a triazole moiety with different alkyl, aryl/*N*-, or, *O*-functionalized alkyl chains as substituents, have also been synthesized by Wang’s group utilizing the Cu(I) mediated click reaction [78]. All of these compounds are gelators of at least one solvent, and eleven compounds are hydrogelators, whereas many of these can congeal aqueous–organic solvents. Of these glycolipids, hydrogelators have been used for the entrapment of toluidine blue dye. In a very recent study, they have also observed that, out of a series of 4,6-*O*-benzylidenated-*d*-glucose (Glc) and *d*-*N*-acetylglucosamine (GlcNAc) containing an N-linked β-1,2,3-triazole moiety having different types of alkyl chains in it, some (**19**, **20**, Figure 15) form gel in oils, including pump and engine oils and also in aqueous DMSO and aqueous EtOH [79]. The phenyl group and the 3-hydroxy group of the sugar unit and the triazolyl moiety containing alkyl chains play an important role during the supramolecular gel-assembly. The gel was characterized by optical microscopy, IR, and rheological studies. Optical microscopy of aqueous DMSO and aqueous EtOH gels, based on two Glc- and GlcNAc-based gelators, showed fibrous network. The mechanism of gel-assembly was established by FT-IR and ^1^H-NMR studies in the sol and gel states. On complete acetylation, the gelation power of these compounds is completely lost.

**Figure 16:**
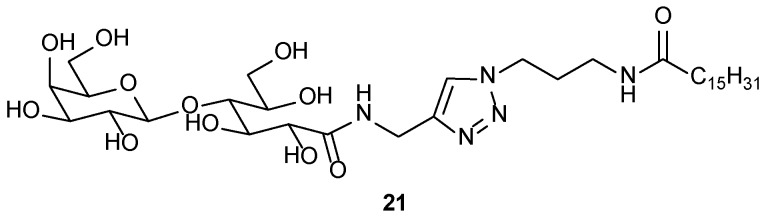
Triazole appended lactobionic acid amide based glycolipid.

Incorporation of a rigid triazole moiety as a spacer through the Cu(I) catalyzed click reaction has also been created for synthesizing a new compound (**21**, Figure 16), derived from the lactobionic acid amide, as polar head group, which gave a shear-induced thixotropic gel [80]. Of these, the hydrogel exhibited interesting behavior due to the polymorphism with respect to the gelator concentration. The gel was analyzed by TEM, cryo-TEM, and SAXS.

**Figure 17:**
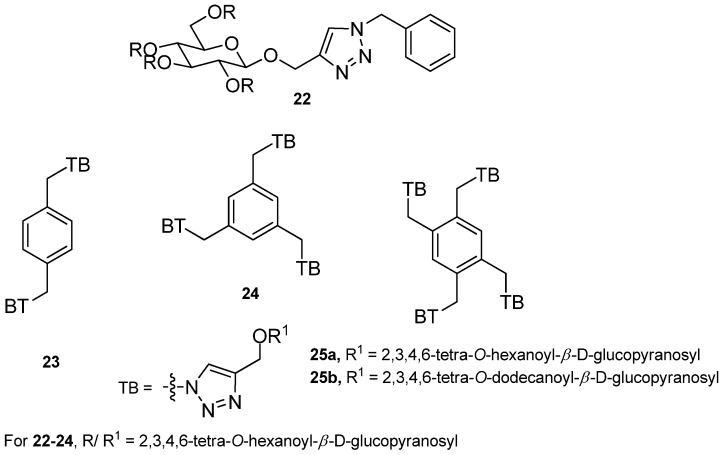
Phenyl centered glycosylated triazoles.

Anchoring glycosylated triazole to the central phenyl ring by Kartha and coworkers [81], has led to the synthesis of several glycolipids, some (**22**–**25**, Figure 17) of which form organogels, particularly in hydrocarbon solvents, even from the water–hydrocarbon biphase. The gelation ability was increased with an increase in the number of substituents (up to tetra substitution) in the central benzene ring. TEM was used to study the morphologies of hexane gels, and molecular modeling was employed to rationalize their self-assembly behavior.

**Figure 18:**
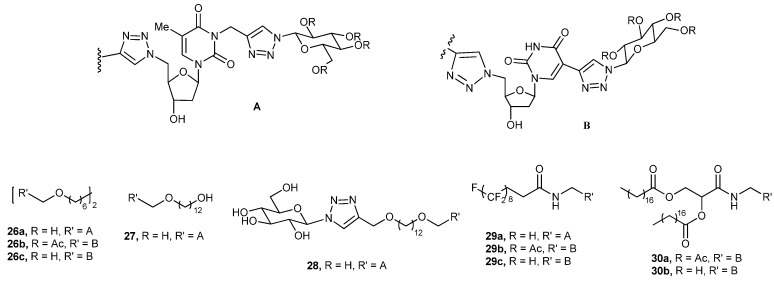
Glycosyl triazole anchored thymidine and uridine based glycolipids.

Native monosaccharide nucleosides like thymidine and uridine have also been considered as a backbone for designing gelator molecules. Barthélémy and coworkers have synthesized several thymidine derived nucleoside symmetric (**26a**) and dissymmetric (**28a**) bolaamphiphiles (which have a thiazole ring attached to the C-5′ of the thimidine unit) (**26a**, **27**, **28a**, and **29a**, Figure 18) [82]. They also prepared a series of uracil-derived glycosyl nucleoside lipids (GNLs) with a triazole moiety attached to the C-5 of uracil utilizing click chemistry (**26b**, **26c**, **29b**, **29c**, **30a**, and **30b**, Figure 18); the series included double-chained compounds, fluorinated single-chain amphiphiles, and bolaamphiphiles [83]. Thymidine derived compounds **26a** and **27** form a hydrogel, but not the dissymmetric bola compound. A TEM image of the hydrogel of **26a** exhibited an anisotropic fibrillar network. The ^1^H-NMR study of the symmetric bola compound (**26a**) in DMSO-d6, and also after gradual addition of H_2_O to the sample, showed gradual upfield shifting of triazole H-atom and thymidine H-5′, indicating involvement of both of these rings in π–π stacking interactions [82]. The hydrogel of **26a** was thixotropic, having a biocompatible rheology, and is useable in a mouse animal model. Most interestingly, the hydrogel allows for the culture of isolated stem cells [82].

Of the six compounds prepared based on the uracil moiety, the two double-chain compounds (**30a** and **30b**) and one (**29b**) of the single-chain compounds form gels in organic solvents. The double-chain U-GNL (**30a**) forms a gel in alcohol, **30b** forms a clear gel in chloroform and an opaque gel in toluene, and **29b** forms an opaque gel in toluene. ATR spectra of the xerogel from the alcohol gel indicates the nonaccessibility of the 3-OH groups to EtOH and a preferential all trans-conformation of the alkyl chains; TEM of the alcogel showed fibrous network [83].

#### 2.2.4. Miscellaneous

**Figure 19:**
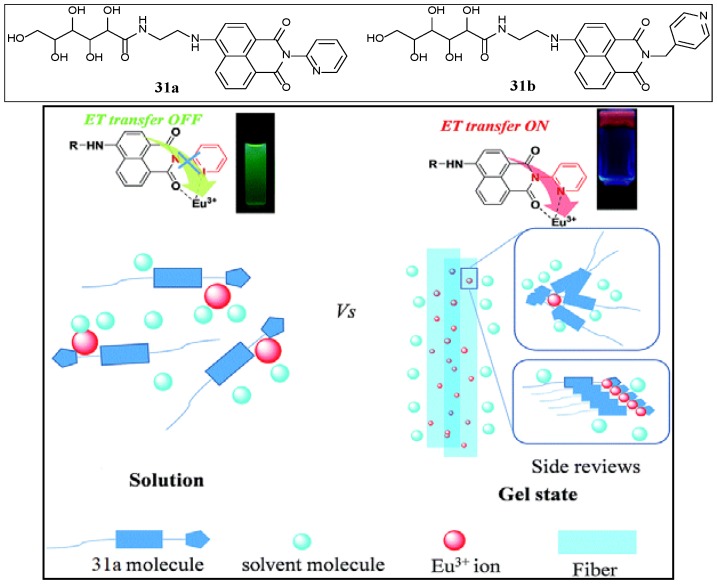
Illustration of the assembly mechanism and energy transfer process of **31a** and Eu^3+^ ions. Reprinted with permission from [84]. Copyright 2015 Royal Society of Chemistry.

Triggered respectively, by ultrasound or by usual heating-cooling process, δ-gluconolactone derived two naphthalimide compounds (**31a** and **31b**, Figure 19), anchored with 2-pyridyl or 4-pyridylmethyl moieties linked with the imide nitrogen, can form gels in organic solvents [84]. In order to understand the gelation process, these researchers have characterized the gels by UV-vis, fluorescence, IR, SEM, and XRD analyses. All these experiments revealed that even a minor variation in the terminal group has a great effect on gelation and ultrasound responsive properties. All these indicated H-bonding, π–π stacking, and hydrophilic interactions, which were responsible for the gel-assembly. Moreover, doping of Eu^3+^ ion on the fresh gel surface, followed by several hours of incubation, resulted in red-emission gels, and indicated the formation of metallogels. Fluorescence experiment of the metallogel and its corresponding sol gave information about the excited energy transfer process in the organogel involved where Eu^3+^ acted as an energy acceptor and the 4-aminonaphthalimide chromophore in the complex metallogel played the role of energy donor (**31a**, Figure 19), and also established that the formation of high-ordered nanofibrils in the metallogel was important during the energy transfer process, as no energy transfer occurred in the sol state. Such findings may pave a new way for the construction of novel rare earth-based luminescent materials.

A tailor-made surfactant, having a disaccharide polar head group, self-assembles in ionic-liquid ethylammonium nitrate (EAN), congealing the latter, forming a thixotropic ionogel with high mechanical strength [85]. Closely-packed bilayers to right-handed twisted ribbons were evidenced in the gel by freeze-fracture transmission electron microscopy (FF-SEM). The gel was also characterized by rheology and tribology experiments, which revealed high mechanical strength and good thixotropy of the gel, a property required for a good lubricant. It is believed that the lubricity of EAN increased due to the presence of the sugar-based gelator in the gel.

**Figure 20:**
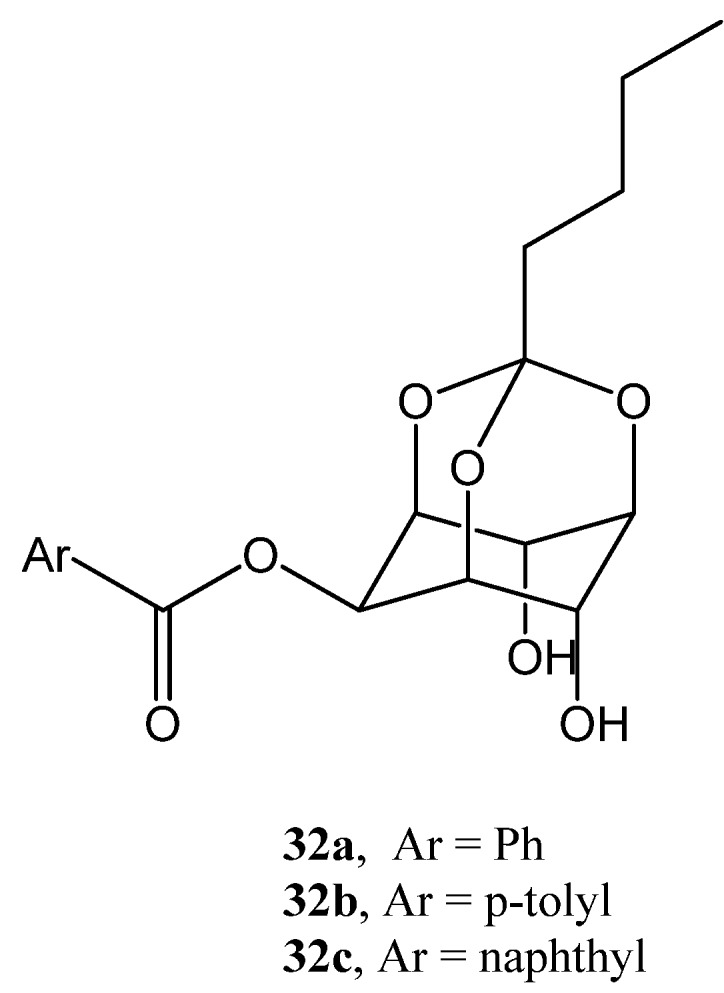
Mono *O*-acyl-*myo*-inositol orthopentanoates.

Sureshan and coworkers used *myo*-inositol as a sugar mimic for the development of a new class of gelators [86]. During rational designing of organogelators, regioselective acylation of *myo*-inositol orthopentanoate furnished several compounds, of which 1,3-benzoate (**32a**), toluate (**32b**), and naphthoate (**32c**, Figure 20) congealed nonpolar solvents and oils. Low CGC and high T_gel_ values indicated these to be stable gels. SEM analysis of the corresponding xerogels exhibited spaghetti-like fibrillar morphology. Interestingly, upon cooling hot solutions of the gelators, which had low-concentration formed crystals, and the other, of high concentration, gave a transluscent gel. XRD of the crystals and XRPD of the xerogel, along with the TGA profiles of both, indicated that intermolecular H-bonding played an important role in both, and additionally, that the involvement of water molecules in the crystals, through which 1D fibrillar layers were interdigitated. The study sheds some light on the concentration-dependent switch of a gelator between a crystal and a gel.

Phytosphingosenes have been used for the synthesis of several organogelators, those having amide and urea linkages, and can congeal alkanes, aromatic oils, vegetable oils, mineral oils, and silicone oils [87].

## 3. Applications

### 3.1. Stimuli Responsive Organogels

**Figure 21:**
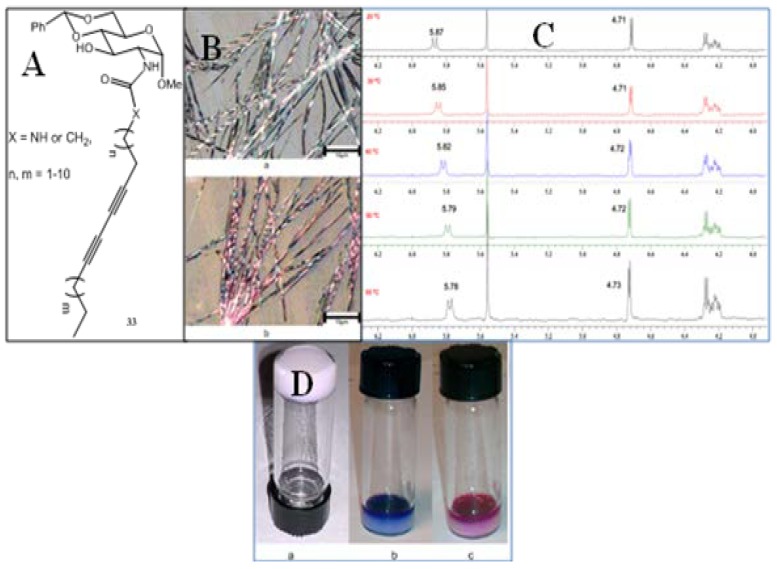
(**A**) Structure of the gelators; (**B**,**C**) optical micrographs of a gel formed by compound **33** (with n = 7, m = 8) in ethanol at 0.8 mg/mL. (a) Before treatment of the gel with UV light. (b) After irradiation with UV light for 1 min. (**C**): Temperature dependent ^1^H-NMR study of the gelator from 20 to 55 °C. Note that the NH bond absorption shifted gradually from 5.87 to 5.78 ppm at 55 °C; (**D**) gel formed by compounds **33** (with n = 7, m = 8) and its responses to UV treatment. (a) An opaque gel formed by this compound in ethanol at 1.5 mg/mL. (b) The gel in vial (**A**) was treated with UV irradiation for 7 min through the top of the vial. (c) The gel in vial (**B**) turned purple-red when heating at 70 °C in a water bath. Reprinted with permission from [99]. Copyright 2015 American Chemical Society.

Supramolecular gels can be sensitized to external stimuli such as temperature, pH, light, or chemicals, to undergo a reversible sol-gel phase transition [5,27,88]. A blue to red color transition in response to environmental changes, and also, in the presence of biomolecules is exhibited by polydiacetylenes (PDAs) [89,90,91,92,93]. PDAs have nonlinear optical properties and show other optical effects [94,95,96,97,98]; these properties make PDAs more attractive for further study in scientific communities. With this in mind, Wang et al. synthesized several glycolipids [99] containing *d*-glucosamine derivatives as the head group, and a long chain containing diacetylene linked to sugar 2-amino nitrogen via amide or urea linkage (**33**, Figure 21). The majority of these glycolipids were organogelators that gelated EtOH, toluene, and aqueous EtOH. Electron microscopy studies showed gels having fibrous/tubular/sheet-like morphologies. One long chain amide, 10,12-tricosadiynoic acid [**33** with n = 7, m = 8, Figure 21], formed polymerizable helices. ^1^H-NMR studies indicated H-bonding to be an important intermolecular force for gel assembly. Ethanol gels obtained from two amides and two ureas were characterized after UV-irradiated polymerization. Curiously, the thermal stability of the cross-linked polydiacetylene (PDA) gels increased, but the morphologies remained apparently similar. The color transitions of such gels upon temperature changes were also investigated using UV-vis spectroscopy; a visible change could be detected. This shows the future applicability of such a gel.

**Figure 22:**
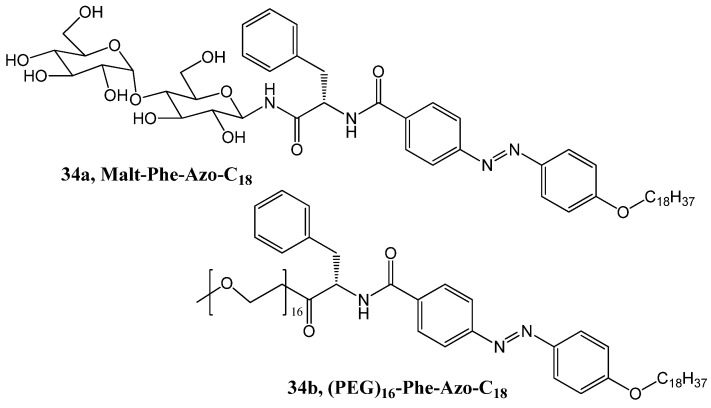
Maltose and PEG derived amphiphiles containing azobenzene.

Oriol and coworkers have designed and synthesized several amphiphilic compounds containing azobenzene as a photosensitive unit, PEG or maltose as the polar head groups and *l*-phenylalanine as the linker [100]. Of these, the maltose derived amphiphile (Malt-Phe-Azo-C_18_, **34a**, Figure 22), and except PEG_16_-, all other PEG-derived amphiphiles were proved to be efficient photoresponsive organogelators. The morphologies of the gels were characterized by electron microscopy, and their chiroptical properties were also studied. Of these, two gels based on PEG_18_-Phe-Azo-C_18_, **34b** (5%, *w*/*w*, in DMSO) and Malt-Phe-Azo-C_18_ (0.5%, *w*/*w*, in 1-dodecanol) could switch reversibly between gel-sol transition, after being irradiated by UV-light at 365 nm; this was followed by thermal back, i.e., sol-gel in the dark. Due to the less hindered trans-cis conversion of the N=N group, the maltose derived gel, in spite of slight modification of UV-vis and Electronic circular dichroism spectra, showed a more efficient photo-induced switch with respect to the other gel.

A series of new gelators based on *d*-gluconic acetal forming multistimuli responsive gels (viz. phase transition by anion, base, ultrasound, and mechanical stress), have been reported. An uncommon anion responsive behavior was observed [101]. Gel disruption by anion was studied by ^1^H-NMR spectroscopic studies. Of the four gels, one exhibited versatile application for waste water treatment, manufacture of optical device, self-healing properties, and spilled oil-recovery.

**Figure 23:**
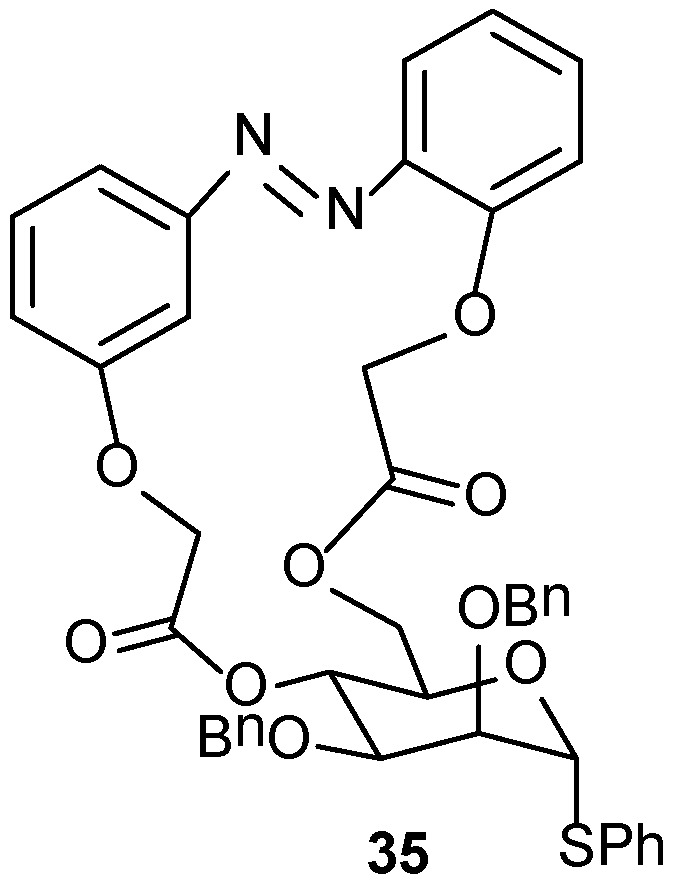
Mannose derived macrocyclic azobenzene.

A one-pot synthesis of sugar derived azobenzene (35, Figure 23) by macrocyclization has been described by Lin et al. [102]. The macrocyclic molecules self-assemble in cyclohexane and EtOH forming stable gels and show stimuli-responsive behavior upon exposure to thermal-, photo- and mechanical-stimuli. The chirality of the sugar unit is transferred to both the E- and Z-geometry of the azo group. The P-configuration of both compounds has been confirmed by CD spectra and also by time dependent-density functional theory (TD-DFT) calculations.

**Figure 24:**
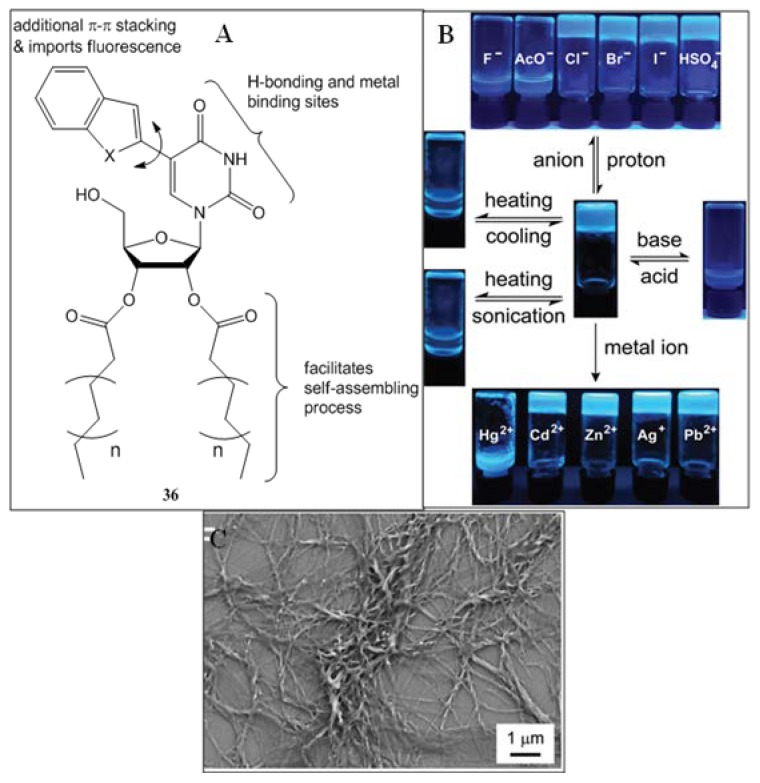
(**A**) Structures of uracil derived glycolipids; (**B**) responsiveness of benzothiophene-modified nucleolipid gel **36** (with a palmitoyl alkyl chain) to physical (temperature and sonication) and chemical stimuli (anion, acid-base, and metal ion). Photographs under UV illumination (365 nm) clearly show the changes in fluorescence upon application of external stimuli; (**C**) field emission scanning electron microscopy (FESEM) images of its xerogels forming twisted fibers. Reprinted with permission from [103]. Copyright 2016 Royal Society of Chemistry.

Srivatsan and coworkers have designed and synthesized several environmentally sensitive fluorescent nucleolipid analogs containing 5-(benzofuran-2-yl)uracil and 5-(benzo[b]thiophen-2-yl)uracil core units as the head groups and attached fatty acid chains (octanoyl/myristoyl/palmitoyl/stearoyl) to the sugar hydroxyl groups through ester linkage as the tail part (**36**, Figure 24A) [103]. Depending on the attached fluorescent probe and the alkyl chains, organogels displaying different morphologies like fibers, twisted/helical ribbons, and nanotubes, were obtained. The gels were characterized by variable temperature ^1^H-NMR spectroscopy, and XRD of crystal and XRPD of the xerogels. Altogether these pointed out that H-bonding, π–π stacking, and hydrophobic effects were responsible for the supramolecular gel assembly. One model gelator based on the anchored benzo[b]thiophen probe, having a palmitoyl alkyl chain, showed aggregation induced enhanced emission. The gelation and photophysical properties are switchable by external stimuli like temperature, ultrasound, and chemicals (Figure 24B). Out of several tested cations and anions, phase transition (gel to sol) was separately evidenced by Hg^2+^ and F^−^ ions. FESEM images of the xerogels of this organogelator exhibited twisted fibers (Figure 24C).

**Figure 25:**
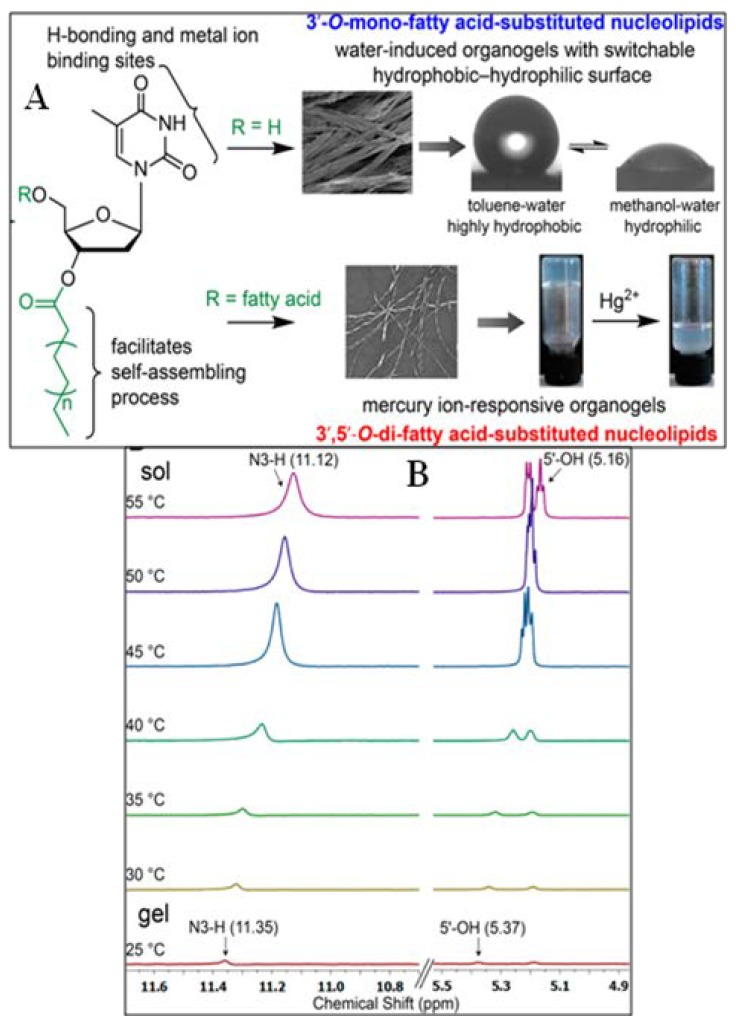
(**A**) Design of self-assembling thymidine nucleolipids, which show different gelation behavior, morphology, surface tunability, and metal-ion responsiveness depending on the site of attachment of the fatty acid acyl chain onto the sugar residue. Water induces the supramolecular gelation of 3′-*O*-monofatty acid-substituted nucleolipids dispersed in organic solvents. The surface of the xerogel films could be tuned between highly hydrophobic and hydrophilic states using an appropriate organic solvent-water mixture. 3′,5′-*O*-Difatty acid-substituted nucleolipids form organogels, which is highly responsive to the presence of Hg^2+^ ions; (**B**) 1H NMR spectra of a 3′-stearoyl derived glycolipid gel (partial gel, d6-DMSO-water = 95:5) at its CGC as a function of increasing temperature. N3-H and 5′-OH atoms exhibited a discernible upfield shift in their proton signals during gel to sol transition. Reprinted with permission from [104]. Copyright 2016 American Chemical Society.

This group has attached various fatty acids at the 3′- or 3′,5′-deoxyribose of the thymidine nucleoside to generate new functional materials capable of metal ion responsiveness and having surface tunability (Figure 25A) [104]. The difatty acid derivatives are organogelators, whereas the 3′-fatty acid derivatives form water induced gels. The nucleolipids having dodecanoyl/myristoyl/palmitoyl/stearoyl chains form gels with DMSO, DMF, MeCN, MeOH, CCl_4_, dioxane, and toluene. ^1^H-NMR of the 3′-stearoyl derived glycolipid clearly established the involvement of N-3H and 5′-OH in an H-bonding interaction during gel assembly (Figure 25B). This along with SEM, XRD, and PXRD also indicate H-bonding, π–π stacking, van der Waals interactions, and hydrophobic effects, which are responsible for gelation. In some cases, gelation is induced by water. Moreover, the distrearic acid-based thymidine nucleolipid-based organogel was highly sensitive to the presence of Hg^2+^ ions (Figure 25A).

Very recently, Jia et al. have synthesized several nucleoside lipids based on uracil, attached at C-5 with N-alkylated (C8-, C12-, and C16-chains) carbazole, and studied the gelation of those [105]. Compounds having C12- and C16-alkyl chains formed stable organogels in toluene-petroleum ether. Interestingly, the organogels emitted blue light, and can act as fluoride ion sensor, as the emission gets destroyed by addition of F^−1^.

### 3.2. Phase Selective Gels and Some Selected Application Toward Oil-Spill Removal and Recovery

Marine oil-spills pollute the environment and are a serious threat to the ecosystem and economy too. There is much interest in developing solidifiers that can tackle such spills [106].

During their study on xylitol acetal and ketals **4a**, **4b**, and **4c** (Figure 3), selective gelation of oil phase by these gelators was observed by Raju et al. [60] from a water–oil biphasic mixture based on different oils; from such organogels recovery of oil was also possible. This shows future applicability of the gelators for oil-spill recovery [60]. Mukhopadhyay and coworkers observed that from a mixture of water-oil, mannose derived organogelators **6a**, **6b**, and **6c** (Figure 5) can form phase-selective oil gels [66,67].

**Figure 26:**
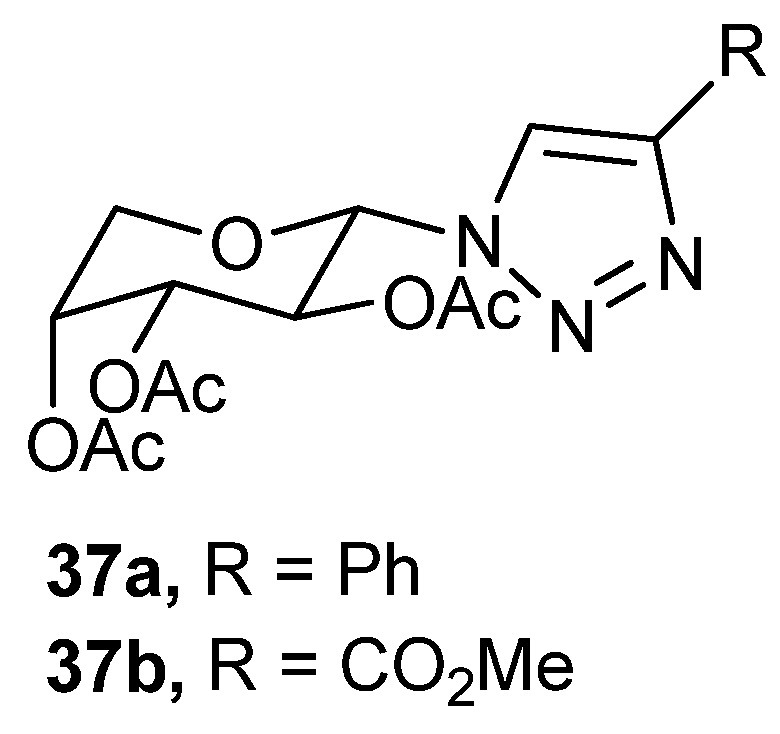
Per-*O*-acetyl-β-*d*-arabinopyranosyl triazoles.

Two triazolyl per-*O*-acetyl-β-*d*-arabinopyranoside with varied substituents (R = Ph, **37a** and R = CO_2_Me, **37b**, Figure 26) on the triazolyl moiety have been synthesized and tested toward gelation in different solvents [107]. These were good gelators of aromatic solvents, including chlorobenzene and fuel oils like petrol, diesel, and kerosene. Gels were characterized using SEM and XRPD (of the xerogels); rheological experiments revealed these gels to be thermoreversible. Fuel–oil phase selective organogelation from aqueous-oil biphasic systems was also tested. The gelators could also congeal crude oils, indicating their potential application for marine and terrestrial oil-spill removal. Some monoglyceride-based organogelators have also been reported to uptake a variety of oils with high uptake capacity from the oil–water mixture [108].

**Figure 27:**
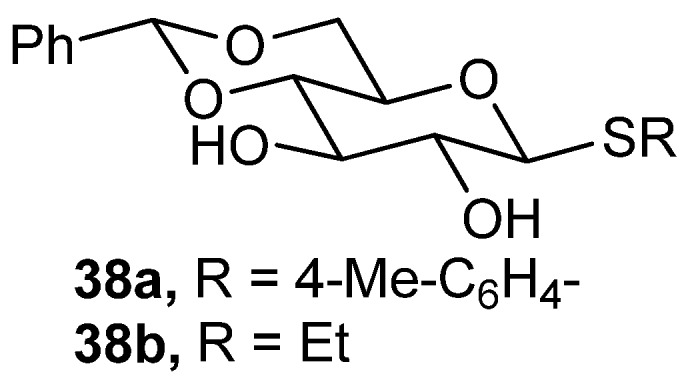
Thioglucoside based organogelators.

Several other organogelators were also previously reported to be capable of selective oil formulation from an aqua–oil biphase [109,110,111,112,113,114,115]. Such selective gelation although works well in small scale in the laboratory, but as far as realistic marine oil-spill recovery is concerned, the above method, except in few cases [114,115], is not a practical one. Sureshan and coworkers have synthesized glucose-based organogelators [116]. Two of these compounds congeal crude oils with CGC 0.5 wt % for **38a** and 0.7 wt % for **38b** (Figure 27). IR and ^1^H-NMR titrations of the gelators in the gelling solvents revealed H-bonding in **38b**, along with π–π interactions and C-H–π interactions, to be the intermolecular interactions playing a crucial role during gel assembly. SEM of the xerogels from benzene gels revealed a fibrous network of the gelator assembly in the corresponding gels. Rheological experiments indicated that the gel from **38a** was more stable than that from gelator **38b**; biphasic CGC of **38a** was slightly higher than that of **38b**. Using gelator **38a**, they have also developed a method that depends upon a real dispersion of fine powder of the organogelators on oil phase with subsequent stable oil-gel formation; the oil-gel on the surface is then scooped out [116]. This may potentially offer a practical solution to marine oil-spill recovery.

Sureshan et al. further developed [117] an efficient method for practical and eco-friendly oil-spill removal and recovery based on a cellulose-organogelator composite. 1,2;5,6-di-cyclohexylidene mannitol (**3a**, Figure 2) was reported earlier to be an efficient organogelator that can form gels in different oils [118]. Surfaces of the cellulose matrix coated with this organogelator become hydrophobic due to the intermolecular H-bonding interaction of the OH of the gelator with cellulose-hydroxy groups; this exposes their hydrophobic parts, making the fibers temporarily hydrophobic. Such gelator-coated cellulose pulp globules have been used for successful oil-spill removal. After being sorbed by oils, the composite cellulose matrix can detach the gelator molecules; these then get dispersed in oil and congeal it, forming the oil-gel. Oil can then be recovered from this gel.

### 3.3. Drug Binding and Their Controlled Release

Several attempts have been made so far to utilize molecular gels for controlled drug release and drug delivery [119,120,121]. Pal and coworkers [57] have utilized sorbitan monostearate (SMS)-sesame oil-based biocompatible organogel in topical drug delivery using metronidazole as a model drug. Good inhibitory action was demonstrated by drug loaded organogels against *E. coli* bacterial colonies. This group has also studied the effect of the concentration of the sorbitan monostearate gelator on mechanical, thermal, and drug release properties of oleogels. A change in gelator (SMS) concentration alters the d-spacing, size of the crystallite, and the lattice strain of the gelator, along with the viscoelastic properties of the oleogels; these in effect, influence drug diffusion and drug release by the gel [58].

Of the 4,6-*O*-benzylidene/alkylidene protected glucosamine derivatives (**8** and **9**, Figure 7), Chen et al. tested one gel in DMSO/H_2_O, formed by the 4,6-*O*-alkylidene protected amide containing cyclohexoyl group on the nitrogen atom (**8** with R = C_6_H_13_), for drug encapsulation and sustained release of chloramphenicol; the gelator concentration and gel strength have a large effect on the sustained release of the drug [70].

Wang’s group used the per-*O*-acetyl maltosyl triazole derivative (**16c** with R = Ph, Figure 13) for the study on drug encapsulation and drug release with the corresponding DMSO-H_2_O-gel [77]; naproxen and chloramphenicol were chosen for drug immobilization in gel and pH dependent drug release. This group has also used a hydrogelator based on a glycolipid (**17** with R = *n*-Pr, Figure 14) for study as a drug (chloramphenicol) carrier and sustained release of the drug [78], and also for the entrapment of toluidine blue dye. Wang’s group has also observed that one (**20** with R = C_6_H_13_, Figure 15) of the GlcNAc-based gelators forms a co-gel in aqueous DMSO with a model drug, naproxen; the controlled drug release in water was also examined by UV-vis spectroscopy [79].

**Figure 28:**
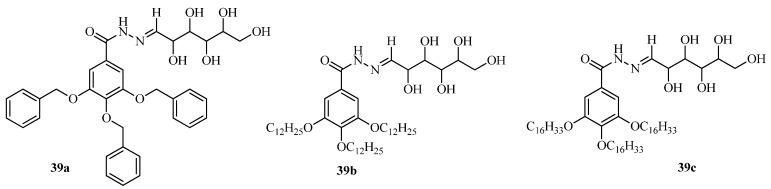
Glucose derived poly(arylether)dendritic molecules.

Glucose derived poly(arylether) dendritic molecules (**39a–c**, Figure 28), exhibit gelation ability; **39b** and **39c** congeal DMSO and **39a** gels in aqueous DMSO (1:1) [122]. The gels have been characterized by SEM, TEM, and PXRD of the xerogel, and also by rheological experiments. The **39a** based gel was cell viable and also tested for entrapment and release of the model hydrophobic dyes Eosin Y and Toluidine blue, along with a local anesthetic (prolicaine hydrochloride). These experiments indicate that the poly(arylether)-based gel made by **39a** can be considered to be an effective drug carrier.

β-Cyclodextrin (β-CD) forms a gel in glycerol solution [123]. The gel acts as a successful drug carrier with 5-fluorouracil or methotrexate. The drug loaded gel has shown better in vitro antitumor efficacy, compared to an anticancer chemotherapeutic, against human hepatocellular carcinoma cells (HepG2 cells). The CD-glycerol gel was characterized using DSC, XRPD (of xerogel), and IR, the last experiment indicating H-bonding to be the main intermolecular interaction for initial fiber formation of the CD-molecules.

### 3.4. Sol-Gel Transcription

**Figure 29:**
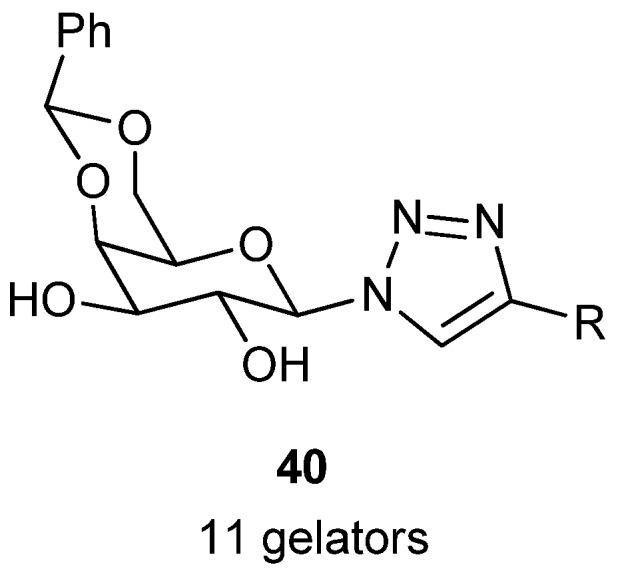
4,6-*O*-Benzylidene-β-*d*-galactopyranosyl triazoles.

Diversified well-defined morphology of supramolecular gels can be used as a template for the transcription of inorganic materials for potential application, such as catalysis, separation, etc. Among these, the formation of a well-defined architecture of nano or meso structured silica by sol-gel transcription of a supramolecular gel has been extensively studied [5,27,124]. A library of eleven organogelators (**40**, Figure 29) has been designed and synthesized utilizing azido 4,6-*O*-benzylidene-β-*d*-galactopyranose as a common building block utilizing click chemistry [125]. Gelators with an alkyl chain on the triazole moiety, gelated alkanes, and tetraethyl orthosilicate (TEOS), those having an aromatic substituent on the triazole moiety, caused congelation of aromatic solvents. The differences in gelation have been exploited for sol-gel transcription (based on **40**, with R = C_12_H_25_), together with synthesis of porous polystyrene (based on **40**, with R = 2-ClC_6_H_4_). This group has also prepared an anthracene anchored analogue of the above gelators for synthesis of a fluorous gel. Concentration dependent IR and ^1^H-NMR of the sols and gels indicated H-bonding induced the initial self-assembly forming fibrils, followed by different courses of secondary assembly giving different gels. Morphologies of the corresponding xerogels were studied by SEM experiments.

**Figure 30:**
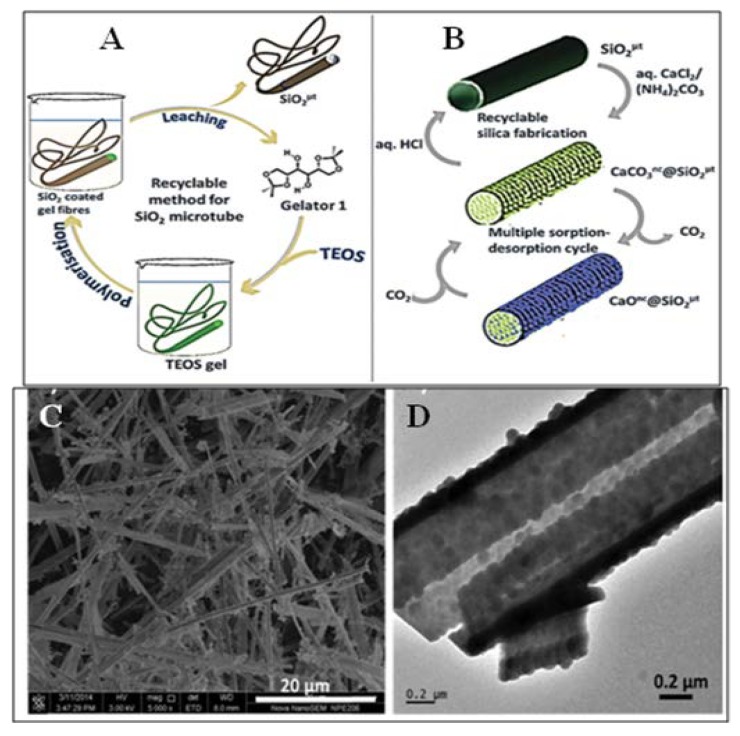
Schematic Proposal for (**A**) large-scale preparation of hollow silica microtubes using recyclable organogel template and (**B**) use of silica microtubes as a platform for growing CaO nanocrystals and their use as sintering-free sorbent for multicycle calcium looping; (**C**) SEM image of silica microtubes after growing CaCO_3_ nanocrystals over their surface; (**D**) TEM image of the CaCO_3_ nc-grown-SiO_2_ microtubes, showing the growth of CaCO_3_ nanocrystals on either surface of the SiO_2_ tubes. Reprinted with permission from [126]. Copyright 2016 Royal Society of Chemistry.

1,2,5,6-Di-*O*-isopropylidene mannitol (**3b**, Figure 2) was reported earlier by Sureshan and coworker [118] to congeal TEOS (Figure 30A), a precursor of silica, and SEM images of the corresponding xerogel revealed the fibrillar morphology of the gelators. This group has also used the gel template for the introduction of typical morphology to the resulting silica during the polymerization of TEOS in the gel state [126]. Instead of using a destructive calcination procedure, they envisioned a more practical large-scalable method for getting transcripted silica microtubes using a recyclable organogel template (Figure 30A). The sol-gel transcripted silica, after washing of the gelator with dichloromethane and methanol, afforded hollow silica tubes with large surface area. The recovered gelator could be reused several times for the preparation of templated silica. Polymerized silica was then characterized by SEM, TEM, SEM-EDAX, PXRD, and IR. They were also able to control the size (diameter) of the hollow tubes (Figure 30B). They have then grown CaCO_3_ nanocrystals on templated silica microtubes (SEM and TEM shown in Figure 30C,D), from which the calcinations then decorated the silica microtubes with CaO nanocrystals at the surface (Figure 30B); they have finally used the resulting sintered-free composite for the efficient uptake and release of CO_2_.

### 3.5. Chirality of Resin

**Figure 31:**
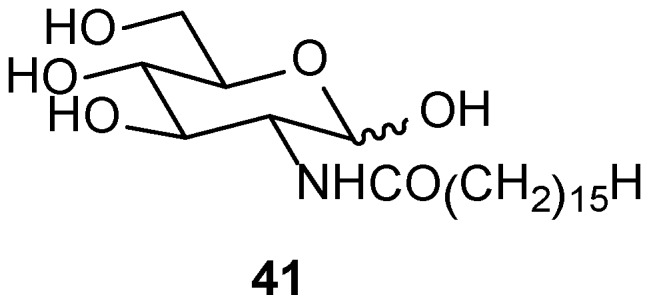
*N*-palmitoylglucosamine.

The inherent chirality of N-palmitoylglucosamine (**41**, Figure 31) induces chirality during its self-assembly in aqueous MeOH, and this property has been utilized as a template for modulation of the chirality of the polymeric resins during their formation based on 3-aminophenol-formaldehyde and also *m*-phenylenediamine-formaldehyde [127]. This causes the formation of left-handed twisted nano-ribbons; the helical pitch decreases with increasing reaction time. Finally, the removal of the template affords resin nanotubes, CD of which indicated the organopolymeric resin-nanotubes to be chiral. Carbonization of such helical 3-aminophenol-formaldehyde resin based nanotubes generated the corresponding M-helical carbonaceous nanotubes. It may be mentioned here that carbonaceous nanotubes and nanofibers have found application in electromagnetic wave absorption and energy-storage [128].

### 3.6. Semiconductor Fabric

**Figure 32:**
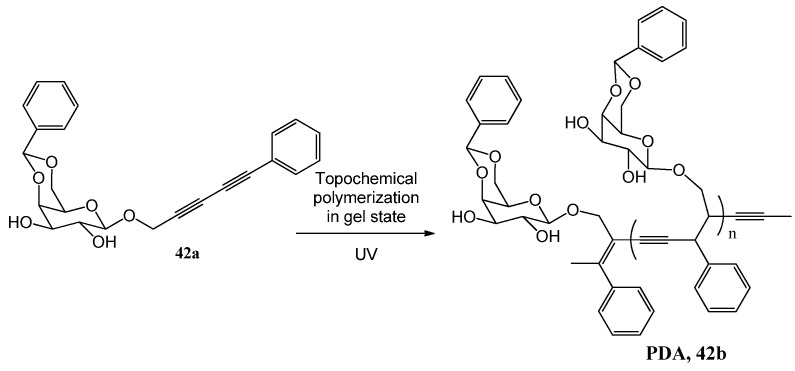
Galactose derived poly diacetylenes.

Earlier, several methods have been employed for making conducting cloths [129,130,131], but these were associated with some serious drawbacks [132,133]. In order to generate conducting cloths with an even distribution of the conducting material throughout the fibers, which are held together tightly, Sureshan and coworkers have designed and synthesized a diacetylene 4,6-*O*-benzylidene-β-*d*-galactopyranoside that congealed different organic liquids [134]. Photoirradiated topological polymerization of the self-assembled gelator molecules (**42a**, Figure 32) in the gel state resulted in formation of poly diacetylenes (PDA, **42b**, Figure 32). Gelation on the fabric followed by photo-polymerization formed the PDA-fabrics. Of these, a benzylidene-free PDA-cotton textile with a semiconducting property exhibited surface resistivity of 10^9^ Ω/sq. This may have a promising future application for the development of semiconducting fabrics.

### 3.7. Lectin Binding

**Figure 33:**
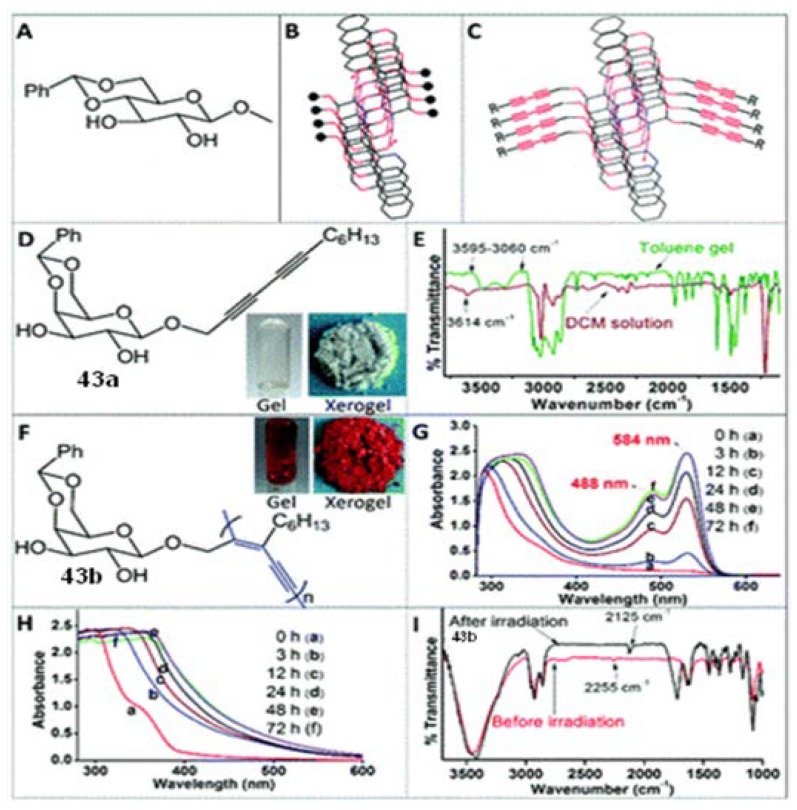
(**A**) Chemical structure and (**B**) packing of 4,6-*O*-benzylidene methyl β-*d*-glucopyranoside in its gel showing a stacked arrangement of methyl groups; (**C**) proposed packing arrangement of the diacetylene functionalized gelator; (**D**) chemical structure of a photo-polymerizable organogelator **43a**. An inverted test-tube image of the toluene gel is also shown; (**E**) IR spectral comparison of DCM solution with the toluene gel of diyne **43a**; (**F**) chemical structure of **43b** obtained by topochemical polymerization of diyne **43a** in the gel state. A photograph of the polymerized gel is also shown; (**G**) and (**H**) time-dependent UV-visible spectra of **43a** in toluene gel and in DCM solution, respectively; (**I**) an overlay of the FT-IR spectra of the xerogels made before (**43a**) and after UV-irradiation of toluene gel (**43b**). Reprinted with permission from [135]. Copyright 2016 Royal Society of Chemistry.

Sureshan and coworkers have designed and synthesized a galactose derived glycoside containing a diacetylene moiety in the aglycon part (**43a**, Figure 33); this compound forms gels in aromatic solvents [135]. IR and ^1^H-NMR indicated H-bonding to be responsible for the gel assembly in the toluene gel. Irradiation (300 nm) of the toluene gel for 2 days converted the transparent colorless gel into an orange-red colored gel, suggesting topochemical polymerization of diacetylene in the gel state generating a polymeric (PDA, **43b**, Figure 33) gel. This was corroborated by the disappearance of a carbon–carbon triple bond band in IR at 2255 cm^−1^, with the concomitant appearance of a new band in IR at 2125 cm^−1^ for a carbon–carbon triple bond stretching in PDA after irradiation. This observation was also supported by the corresponding Raman spectroscopy. A glass slide was then coated with a toluene gel, which, after irradiation (300 nm) for 7 days, turned into a red film, thus giving a glass plate coated with a PDA-based glycopolymer. The glass slide was then dipped into a diluted HCl solution to remove the 4,6-*O*-benzylidene protecting group from the D-Gal unit. After washing the slide with water, it was finally washed with phosphate-buffered saline. The resulting plate (PDA-a) was then used for the binding study with three β-*d*-galactopyranoside specific lectins; lectin binding was also compared to the plate coated with methyl β-*d*-galactopyranoside. These revealed a 1000 fold more effective lectin binding of the plates with PDA-a coat. Efficacy-wise this method was superior to the traditional methods of synthesizing a multivalent surface [136]. Thus, this method can have great potential for the development of diagnostic tools for the detection of diseases involving lectin-binding processes.

### 3.8. Self-Healing Property

The mannose derived organogelators **6a**, **6b**, and **6c** (Figure 5), developed by Mukhopadhyay and coworkers, exhibit self-healing properties, with **6a** showing excellent healing [66,67].

**Figure 34:**
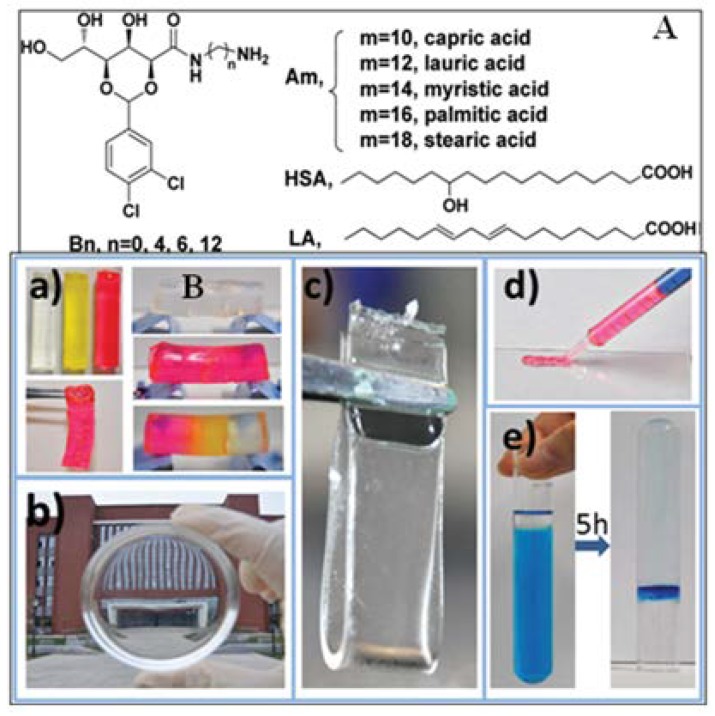
(**A**) Structure of Bn and aliphatic acids; (**B**) (a) Illustration of self-healing properties of the CB gels (2.0% *w*/*v*) obtained from undoped B6–A10 gel, yellow dye-doped B6–A14 gel, and red dye-doped B6–A18 gel. (b) View of the building from a distance of 50 m through B6–A18/CB gel. (c) B6–A18/CB gel film. (d) Extrusion of red dye-doped B6–A18/CB gel from a syringe. (e) 20 mg B6–A18 powders were added to the mixture of 1 mL benzene and 10 mL of 0.1 mM aqueous solution of methylene blue. Reprinted with permission from [137]. Copyright 2016 Royal Society of Chemistry.

Two component gel systems with multifunctional properties, like tunable viscoelasticity and self-healing properties, have been developed by Liu et al. using 2,4-*O*-(3,4-dichloro)benzylidenated-*d*-gluconamide (**44**, Figure 34A) (prepared from long chain diamines) and fatty acids based on acid–base interactions [137]. The gelation ability of the two component gelators (1:1) is dependent on the structure of the components. Of these, B6-Am was a supergelator with CGC <0.1% in chlorobenzene (CB) and *o*-xylene. B6-Am/CB-gel and B6-A18/CB-gel undergo rapid self-healing even after vigorous stirring (Figure 34B(a–d)). Powder of the two component gelators can gel aromatic solvents and can congeal a solvent–water biphasic mixture. It can also absorb a model dye efficiently from the biphasic mixture (Figure 34B(e)).

Zwitterionic copolymer (PMB) containing a fixed benzoxaborole group and zwitterionic glycopolymers (PMG) with varied amounts of sugars have been used to give pH responsive zwitterionic hydrogels [138]. Those with higher sugar content and higher pH were found to have higher strength. The zwitterionic gels were biocompatible; some selected hydrogels were injectable and had self-healing properties.

### 3.9. Structuring Agent

**Figure 35:**
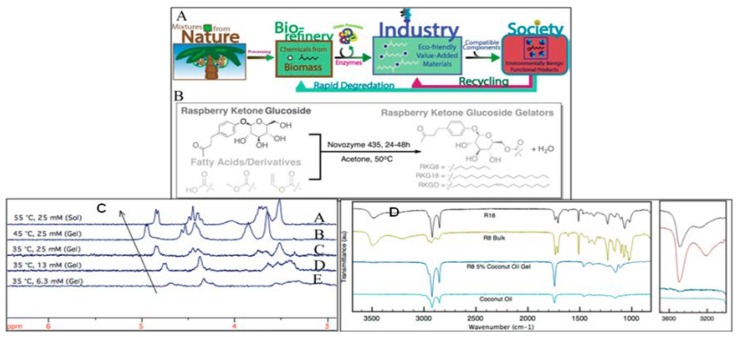
(**A**) Schematic demonstration of the combined biorefinery model with green chemistry principles, extracting reagents from waste and biomass resources to develop functional materials from value-added chemicals; (**B**) synthetic scheme for the development of novel medium- and long-chain triglyceride gelators following the model; (**C**) 1H NMR spectra of RKG8 mixtures in toluene: a close-up on the shifting carbohydrate peaks. The bottom spectra (B–E) are in the gel state, and the top spectrum (A) is a solution. There is a shift in the pyranose hydrogen at the C1 position δ 4.9 following the stretched hydrogen bond in the gel that then relaxes when the molecules are in solution.; (**D**) (Left) FTIR spectra of oleogel and bulk gelators samples; (right) hydrogen bonding region displays weak O–H peaks in the gel spectrum. Reprinted with permission from [139]. Copyright 2015 American Chemical Society.

Due to the benefits to human health, development of oil-structuring agents for use as substitutes of saturated and trans-fats has been growing in interest. In order to develop value-added materials, sugar-based ester organogelators were envisioned in the light of green principles and synthesized (Figure 35A) by John and coworkers using a biocatalyst [139]. Raspberry ketone glycosides were esterified using Novozyme 435 in acetone with an unsaturated medium and long chain fatty acids, and two saturated fatty acids giving the corresponding ester amphiphiles caprylate 9RKG80 and the corresponding stearate (RKG18), along with the monosaturated raspberry ketone glycoside oleate (RKGO); the gel based on the latter gelator showed much higher MGC compared to the other two types. Some of such sugar esters self-assembled in organic solvents and also in edible oils. IR and ^1^H-NMR analyses of the gelator RKG8 (Figure 35B) in toluene gel, as well as in sol states, and its subsequent IR analyses in bulk, in coconut oil gel, and sol states (Figure 35C), revealed H-bonding to be the main noncovalent interaction of the supramolecular gel-assembly. SEM images of the xerogels obtained from the coconut oil gel (after extraction in hexane) of gelator R8 displayed a fibrous network and that of the xerogel from toluene gel exhibited sheet-like ribbons of the gelator assembly. A comparison of the X-ray powder diffraction (XRPD) of the xerogels indicated that gel packing of the fatty acid chain was similar to that of the natural fats.

Chen et al. have studied emulgels based on β-carotene enriched zein-based oil-in-glycerol (O/G). Due to the hydrophobic interaction of zein and β-carotene, an increase of the latter in such emulgels strengthens the gel network, improving their spreadability in the emulgels [140]. This also results in a higher UV-stability of β-carotene; cakes have also been prepared as an alternative to margarine, using zein-based O/G emulgels.

Using Novozyme 435 as catalyst, mannitol was esterified in high yield with octanoic acid in a acetone–hexane mixed solvent medium. The generated mannitol-1,6-di-*O*-octanoate forms a gel in edible oils [141]. The biodegradable, tailor-made sugar-lipid has great potential toward its use as structuring agent for edible oils.

Sunflower oil was reported to be structured by commercial monoglycerides (15% *w*/*w*) and phytosterol (5% *w*/*w*) in the oil dispersion of the mixture [142]. The resulting oleogel was characterized by the usual microscopic, rheological, and calorimetric experimental techniques. The organogel has been used as part substitute of the animal fat for preparation of the frankfurter style sausage, showing new direction in processed foods with the reduction of saturated animal fat.

### 3.10. Medium for Enzyme Catalysis

Organogel medium has an application for carrying out enzyme-catalyzed transformation. In a previous study, 2,3:4,5-*O*-diisopropylidene-β-*d*-fructopyranose has been esterified with palmitic acid in heptanes by passing the mixture continuously through a bed containing CalB lipase enzyme immobilized in microemulsion-based organogels [143], prepared following the reported methods [144,145].

## 4. Conclusions and Future Perspectives

In this review we have focused mainly on carbohydrate-based organogelators and the corresponding gels, and also on their application in various fields, published since 2015. The discovery of gels was a serendipitous phenomenon. But, during the last two to three decades systematic research on molecular gels, in general, and organogels, in particular, has flourished into multidimensional aspects, in terms of the fundamental perspective through which the logical design of new gelators is focused on their multifarious applications. In spite of many research reports in the literature in this field there is still scope for the exploration of other new aspects along with their application in real life, and also to quest for a definite answer to the question ‘why, when, and how do gels form?’. Very recently [146], Weiss in a ‘concept article’ published in this journal, addressed this issue; although he has raised some pertinent general questions regarding the mode of research in this field, but put forward some valuable advice too on ideal methodologies, those should be used for the studies on gels and toward the advancement of the technology; both of which will benefit people working in the field as well as those choosing to begin their research in this field.

## Figures and Tables

**Table 1:** Gelation abilities of compounds **4a–c** in different hydrocarbon solvents ^a^.

Solvent System	MGC (% *w*/*v*) of 4a	MGC (% *w*/*v*) of 4b	MGC (% *w*/*v*) of 4c
Hexane	P	P	P
Octane	P	P	P
Dodecane	1.8	1.91	P
Hexadecane	1.65	1.75	1.9
Benzene	1.16	1.22	1.43
Toluene	1.12	1.2	1.37
Xylene	1.05	1.2	1.35
CRN	1.07	1.28	P
SRN	1.31	P	P
Kerosene	0.62	1.1	1.84
Diesel	0.43	0.62	0.77
Crude oil	1.69	1.85	2.01
Vegetable oil	0.65	0.78	0.92

^a^ MGC = Minimum Gelation Concentration (amount in gram of gelator required for 100 mL of hydrophobic material to be gelated), P = Precipitate, CRN = Cracked naphtha, SRN = Straight run naphtha. Reproduced with permission from a previous paper [60]. Copyright 2017 Royal Society of Chemistry.
